# Supplementary material for: Expression of Interferon Regulatory Factor 8 (IRF8) and Its Association with Infections in Dialysis Patients
Source: Cells. 2023 Jul 19;12(14):1892. doi: 10.3390/cells12141892 (PMC10378315; doi:10.3390/cells12141892)
Supplement: Supplementary file 1 [file cells-12-01892-s001.zip › Supplementary Tables S1 & S2.pdf]

## Supplementary Tables

**Supplementary Table S1.** Assessment of the relationship between duration of kidney replacement therapy and distribution of immune cell subsets.

| Variable | Spearman r | p value |
|---|---|---|
| mDC1 | 0.07 | 0.53 |
| mDC2 | 0.14 | 0.23 |
| pDC | -0.07 | 0.56 |
| NK cells | 0.17 | 0.14 |
| classical monocytes | 0.10 | 0.36 |
| intermediate monocytes | 0.07 | 0.56 |
| nonclassical monocytes | -0.07 | 0.52 |
| CD4+ cells | 0.09 | 0.44 |
| CD8+ cells | -0.12 | 0.3 |
| CD19+ cells | -0.0008 | 0.99 |
| Percent IRF8 positive mDC1 | 0.11 | 0.33 |
| gMFI of IRF8 in mDC1 | 0.09 | 0.45 |
| Percent IRF8 positive mDC2 | 0.03 | 0.83 |
| gMFI of IRF8 in mDC2 | 0.06 | 0.59 |
| Percent IRF8 positive pDC | 0.22 | 0.06 |
| gMFI of IRF8 in pDC | 0.22 | 0.052 |
| Percent IRF8 positive classical monocytes | 0.13 | 0.25 |
| gMFI of IRF8 in classical monocytes | 0.19 | 0.1 |
| Percent IRF8 positive intermediate monocytes | 0.13 | 0.26 |
| gMFI of IRF8 in intermediate monocytes | 0.08 | 0.47 |
| Percent IRF8 positive nonclassical monocytes | 0.16 | 0.17 |
| gMFI of IRF8 in nonclassical monocytes | 0.14 | 0.23 |

IRF8, interferon regulatory factor 8; mDC, myeloid dendritic cells; NK, natural killer; pDC, plasmacytoid dendritic cells.

**Supplementary Table S2.** Assessment of the relationship between causes of renal failure and distribution of immune cell subsets.

| | **Previous renal transplantion n=15** | **Diabetic glomerulosclerosis n=16** | **Chronic glomerulonephritis n=14** | **Nephrosclerosis n=8** |
|---|---|---|---|---|
| **Variable** | **p value** | **p value** | **p value** | **p value** |
| mDC1 | 0.69 | 0.75 | 0.41 | 0.66 |
| mDC2 | 0.94 | 0.97 | 0.08 | 0.57 |
| pDC | 0.83 | 0.55 | 0.64 | 0.78 |
| NK cells | 0.81 | 0.21 | 0.65 | 0.73 |
| classical monocytes | 0.54 | 0.8 | 0.14 | 0.81 |
| intermediate monocytes | 0.13 | 0.26 | 0.65 | 0.19 |
| nonclassical monocytes | 0.58 | 0.69 | 0.74 | 0.96 |
| CD4+ cells | 0.62 | 0.59 | 0.75 | 0.14 |
| CD8+ cells | 0.65 | 0.73 | 0.68 | 0.20 |
| CD19+ cells | 0.87 | 0.43 | 0.57 | 0.08 |
| Percent IRF8 positive mDC1 | 0.76 | 0.73 | 0.91 | 0.25 |
| gMFI of IRF8 in mDC1 | 0.46 | 0.19 | 0.47 | 0.66 |
| Percent IRF8 positive mDC2 | 0.8 | 0.18 | 0.64 | 0.61 |
| gMFI of IRF8 in mDC2 | 0.78 | 0.15 | 0.47 | 0.37 |
| Percent IRF8 positive pDC | 0.90 | 0.87 | 0.16 | 0.77 |
| gMFI of IRF8 in pDC | 0.97 | 0.87 | 0.17 | 0.79 |
| Percent IRF8 positive classical monocytes | 0.52 | 0.84 | 0.29 | 0.80 |
| gMFI of IRF8 in classical monocytes | 0.38 | 0.99 | 0.23 | 0.94 |
| Percent IRF8 positive intermediate monocytes | 0.07 | 0.44 | 0.7 | 0.60 |
| gMFI of IRF8 in intermediate monocytes | 0.12 | 0.37 | 0.83 | 0.64 |
| Percent IRF8 positive nonclassical monocytes | 0.11 | 0.34 | 0.32 | 0.60 |
| gMFI of IRF8 in nonclassical monocytes | 0.25 | 0.39 | 0.40 | 0.46 |

IRF8, interferon regulatory factor 8; mDC, myeloid dendritic cells; NK, natural killer; pDC, plasmacytoid dendritic cells.
