# Supplementary figures and images for: Expression of Interferon Regulatory Factor 8 (IRF8) and Its Association with Infections in Dialysis Patients
Source: Cells. 2023 Jul 19;12(14):1892. doi: 10.3390/cells12141892 (PMC10378315; doi:10.3390/cells12141892)

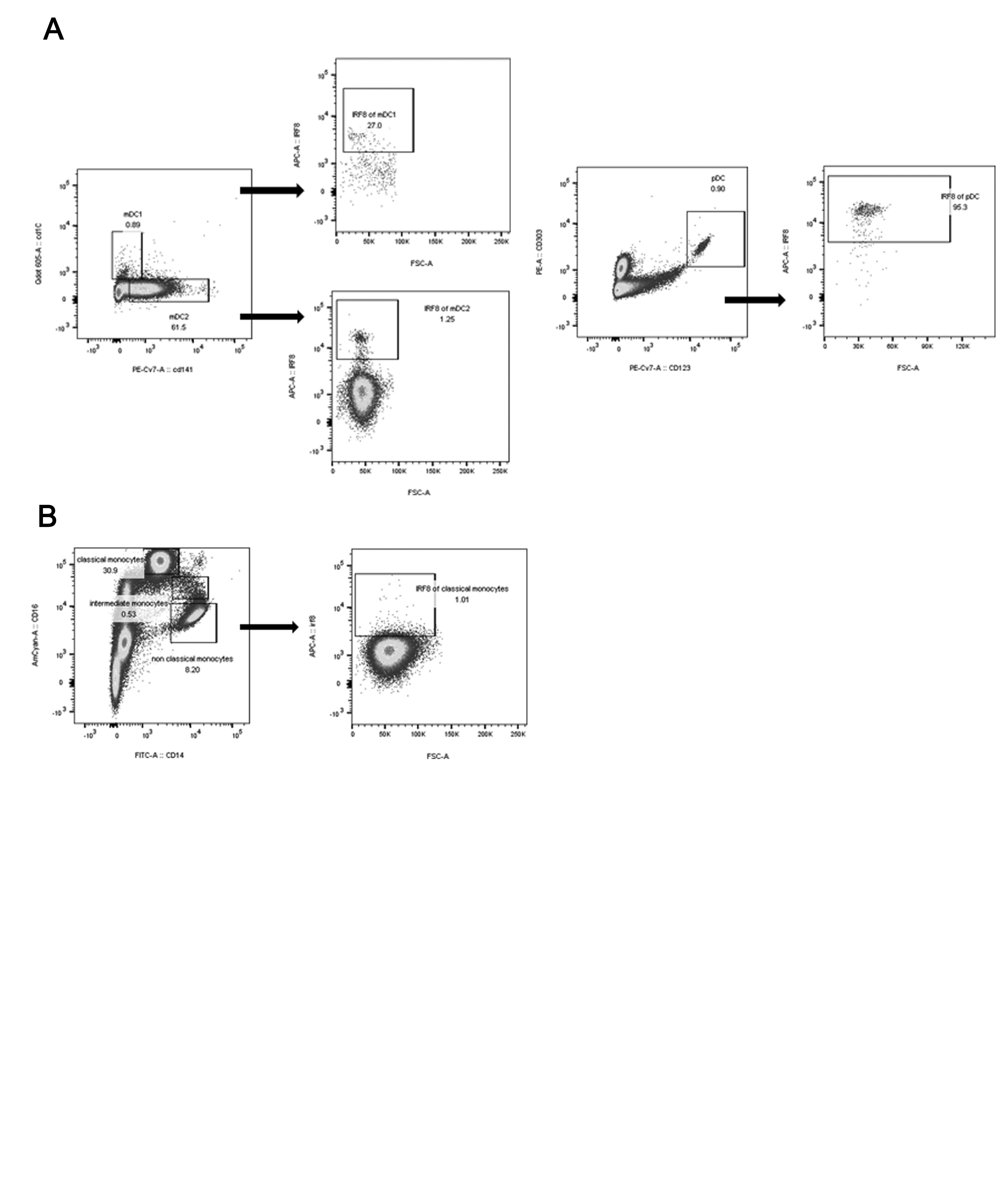

Supplement: Supplementary file 1 [file cells-12-01892-s001.zip › Supplementary Figure S1.png]

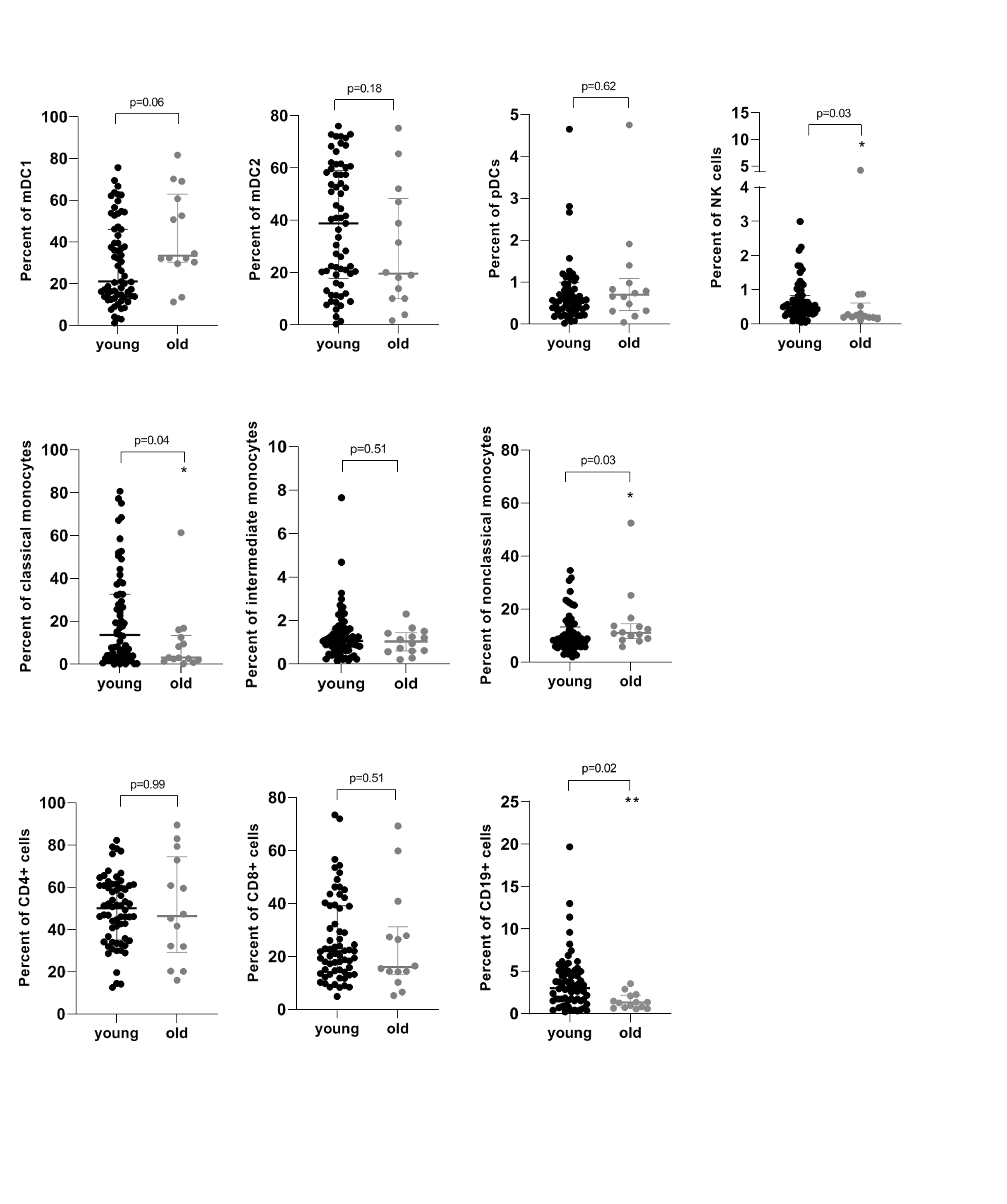

Supplement: Supplementary file 1 [file cells-12-01892-s001.zip › Supplementary Figure S2.png]

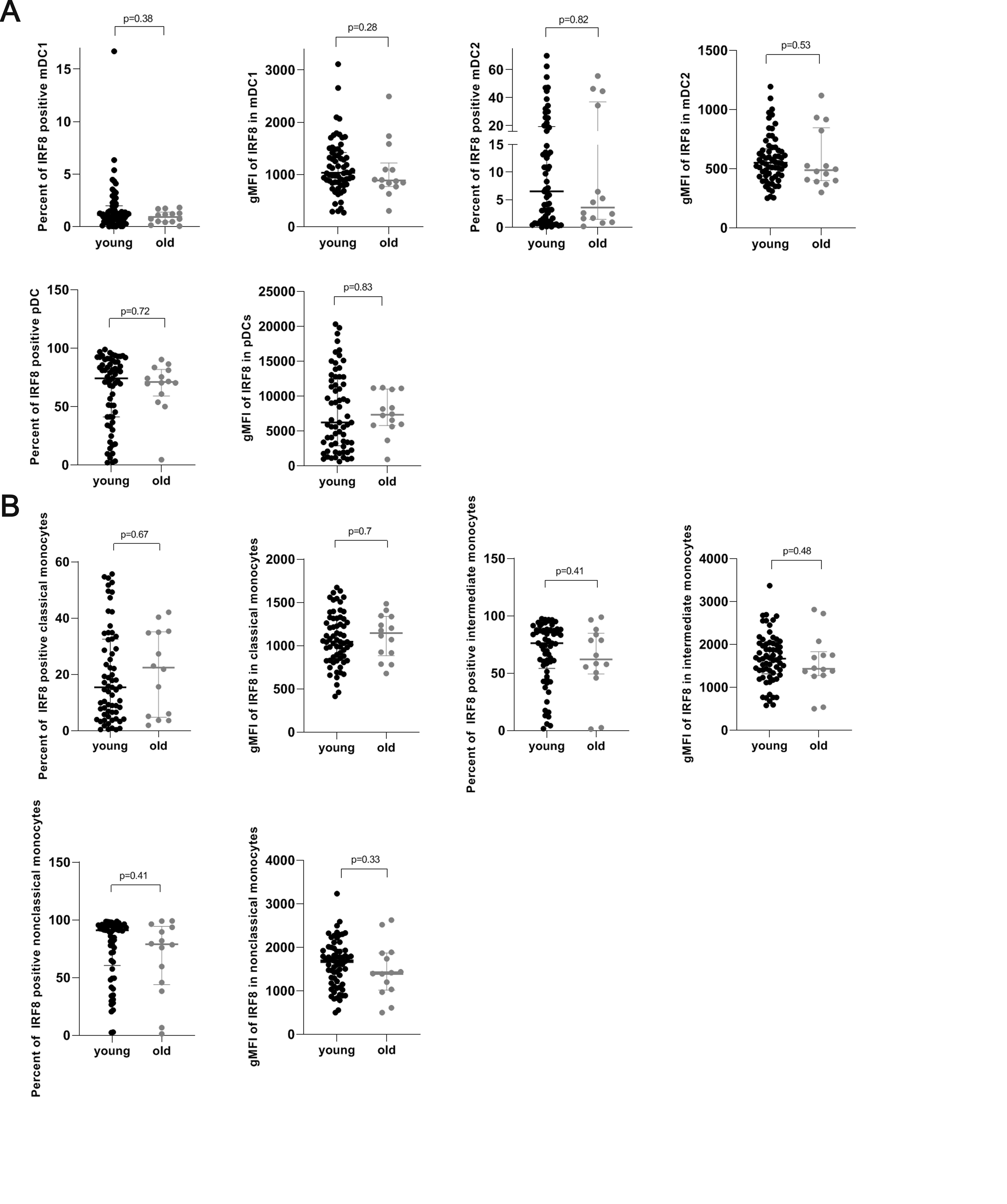

Supplement: Supplementary file 1 [file cells-12-01892-s001.zip › Supplementary Figure S3.png]
